# Prevalence of Myofascial Trigger Points in Patients with Radiating and Non-Radiating Low Back Pain: A Systematic Review

**DOI:** 10.3390/biomedicines13061453

**Published:** 2025-06-12

**Authors:** Germán Monclús-Díez, María José Díaz-Arribas, César Fernández-de-las-Peñas, Dariusz Kosson, Marcin Kołacz, Mateusz D. Kobylarz, Sandra Sánchez-Jorge, Juan Antonio Valera-Calero

**Affiliations:** 1 Department of Radiology, Rehabilitation and Physiotherapy, Faculty of Nursery, Physiotherapy and Podiatry, Complutense University of Madrid, 28040 Madrid, Spain; gmonclus@ucm.es (G.M.-D.); mjdiazar@ucm.es (M.J.D.-A.); 2 Grupo InPhysio, Instituto de Investigación Sanitaria del Hospital Clínico San Carlos (IdISSC), 28040 Madrid, Spain; 3 Department of Physical Therapy, Occupational Therapy, Rehabilitation and Physical Medicine, Universidad Rey Juan Carlos, 28922 Alcorcón, Spain; cesar.fernandez@urjc.es; 4 Department of Anaesthesiology and Intensive Care, Division of Teaching, Medical University of Warsaw, 02-005 Warsaw, Poland; dariusz.kosson@wum.edu.pl; 5 1st Department of Anaesthesiology and Intensive Care, Medical University of Warsaw, 02-005 Warsaw, Poland; marcin.kolacz@wum.edu.pl; 6 Escuela Internacional de Doctorado, Universidad Rey Juan Carlos, 28922 Alcorcón, Spain; md.kobylarz@alumnos.urjc.es; 7 Faculty of Health Sciences, Universidad Francisco de Vitoria, 28223 Madrid, Spain

**Keywords:** low back pain, myofascial trigger points, prevalence

## Abstract

**Background/Objectives:** Muscle tissues are a common source of symptoms related to low back pain (LBP), with myofascial trigger points (MTrPs) being a significant contributor. Since previous meta-analyses support interventions targeting MTrPs for reducing pain and improving functional disability in patients with LBP, this review aimed to synthesize current knowledge on the prevalence of MTrPs in LBP patients. **Methods**: To conduct this systematic review, data were collected from PubMed, Cochrane, and Web of Science. Published articles at any time up to February 2025 that comprised descriptive, observational, or experimental studies in English/Spanish language reporting the prevalence of active or latent MTrPs in patients with LBP were eligible. After assessing the methodological quality, a structured and qualitative synthesis was conducted using a standardized form that captured participant characteristics, evaluated muscles, the number or percentage of active and latent MTrPs in each group, clinical features, summarized results, and conclusions. **Results**: Nine articles with acceptable methodological quality were included. The prevalence of active MTrPs in patients with LBP was quadratus lumborum (ranging from 30% to 55%), gluteus medius (from 34% to 45%), piriformis (42%), psoas (from 5% to 10%), and lumbar iliocostalis (from 33% to 38%). Latent MTrPs were most common in the gluteus medius (74%) and quadratus lumborum (14–17%), with the piriformis, psoas, and lumbar iliocostalis also affected. **Conclusions**: Active and latent MTrPs are common in muscles such as the quadratus lumborum, gluteus medius, and iliocostalis in individuals with LBP, with prevalence varying by pain chronicity and etiology. MTrPs in the gluteal region are more frequent in lumbosacral radiculopathy, suggesting a neurogenic-like component. Since the subjectivity of manual palpation and study heterogeneity limit generalizability of the results, future research should standardize diagnostic criteria of MTrPs to ensure the consistency of results.

## 1. Introduction

Low back pain (LBP) is a pain condition that can be associated with various pathologies [1] and is defined as a painful sensation localized in the posterior region of the trunk, situated between the lower margin of the last ribs and the inferior gluteal folds, with or without radiation to one or both lower extremities, and persisting for at least one day [2,3]. It encompasses a spectrum of pain phenotypes, including nociceptive, neuropathic or radicular pain, and, in some cases, nociplastic pain, which is attributed to amplification of pain within the central nervous system [4,5].

LBP is currently classified as specific and non-specific, depending on its underlying cause. Specific LBP is associated with a clear pathoanatomical cause, which may stem from damage affecting structures beyond the lumbar spine, specific spinal disorders (e.g., epidural abscess, compression fractures, spondyloarthropathies, neoplasms, and cauda equina syndrome) or other medical conditions causing radiculopathy or spinal canal stenosis [6]. Although these are clinically significant conditions, prevalence studies estimate that only 5% of LBP cases are attributable to such specific causes [7]. Approximately 4% involve compression fractures, 3% involve spinal stenosis, and less than 1% are due to conditions such as tumors or infections [7]. To identify cases of LBP with potentially serious causes, red flags (e.g., nocturnal pain or unexplained weight loss) are used, although individual signs typically have low diagnostic accuracy [8]. Consequently, a more effective diagnostic approach is to consider a combination of clinical features to determine when further investigation is warranted.

In contrast, most patients with LBP (up to 90%) lack an identifiable pathoanatomical cause [9] and are classified as non-specific. In such cases, while various lumbar structures, such as intervertebral disks and facet joints, are plausible pain sources, clinical tests cannot reliably attribute pain symptoms to any particular structure [10,11]. However, muscles are a frequent source of symptoms associated with LBP [12]. Myofascial trigger points (MTrPs) are defined as “hyperirritable spots in skeletal muscle, associated with a palpable, hypersensitive nodule within a taut band. These areas are painful upon manual compression and can generate characteristic referred pain, referred tenderness, motor dysfunction, and autonomic phenomena” [13]. MTrPs are considered one of the most common sources of symptoms associated with non-specific LBP [14,15] and an important therapeutic target for improving pain, disability, and quality of life in these patients [16,17].

Although the exact pathophysiology of MTrPs remains unclear, studies suggest that nociceptive stimuli from MTrPs are partly induced by muscle ischemia and hypoxia resulting from capillary compression by the taut bands. The low oxygen levels in blood lead to pH reduction (acidification), activating acid-sensitive ion channels, inhibiting acetylcholinesterase, and promoting the release of ATP, bradykinin, tumor necrosis factor-alpha, interleukins, serotonin, norepinephrine, substance P, and calcitonin gene-related peptide [18].

Clinically, MTrPs are classified as active or latent. Active MTrPs are responsible for persistent pain that reproduces the patient’s symptoms and, when stimulated, elicit a response the patient recognizes as part of their symptomatology. They are also associated with functional limitations, restricted range of motion, and muscle strength deficits. Latent MTrPs, on the other hand, are clinically silent, as they do not cause spontaneous pain. However, they are sensitive to palpation and, while they do not reproduce the patient’s typical symptoms, they contribute to increased muscle tension and restricted range of motion [19].

Although individual studies have documented that MTrPs occur in a large proportion of patients with non-specific LBP [12,20], the true scope of this problem remains unclear. Although previous reviews on the prevalence of MTrPs in spinal pain disorders were conducted over a decade ago [12,21], none focused specifically on LBP nor distinguished between clinical presentations of LBP (with or without lower-limb referred pain). Moreover, a thorough MTrP examination has been shown to require at least 15 min when assessing all major muscle groups (which is a timeframe that can limit feasibility in busy clinical settings) [22]. Without knowing which lumbar and adjacent muscles most commonly harbor active MTrPs, clinicians may waste valuable time palpating low-yield sites. Establishing the pooled prevalence of MTrPs in non-specific LBP is therefore essential not only to justify their diagnostic and therapeutic priority, but also to optimize the examination workflow (by first targeting the most frequently affected muscles, practitioners can improve efficiency, reproduce patient symptoms more quickly, and only then expand to less common locations if needed). Failure to recognize both the magnitude and the practical examination burden of MTrPs may perpetuate misdiagnosis, delay appropriate management, and contribute to prolonged pain, disability, and healthcare costs. Therefore, the objectives of this review were to conduct a qualitative synthesis on the prevalence of MTrPs in patients experiencing LBP and to identify which muscles are the most commonly affected.

## 2. Materials and Methods

### 2.1. Study Design

A systematic literature review was conducted between September 2024 and April 2025, adhering to the Preferred Reporting Items for Systematic Reviews and Meta-Analyses (PRISMA) guidelines [23]. The study protocol was prospectively registered on the Open Science Framework platform (identifier DOI: 10.17605/OSF.IO/7AJHP).

### 2.2. Search and Article Management Resources

In alignment with current recommendations for systematic reviews, which suggest consulting at least three databases for comprehensive coverage [24], literature searches were conducted in PubMed, Cochrane, and Web of Science. Additionally, references from identified articles were reviewed. Since not all journals are indexed in these databases, a manual search was performed for key journals.

The Mendeley Reference Manager (v2.129.0) was used to manage search results, providing efficient organization of references by topic, document type, and priority. This tool facilitated citation, document analysis, and the elimination of duplicate studies using the “check for duplicates” feature [25].

### 2.3. Search Strategy

The literature search was conducted using a combination of Medical Subject Headings (MeSH) descriptors and free-text terms, structured around the PICO framework (Population, Intervention, Comparison, and Outcome):

- Population: Individuals with a diagnosis of non-specific LBP (no underlying medical condition) with or without radiating pain to the lower extremity.
- Intervention: No applicable.
- Comparison: Asymptomatic individuals or comparisons between painful or dominant sides.
- Outcome: Diagnosis of MTrPs, active or latent with manual palpation and adhered the latest Delphi consensus guidelines [13,26].

The search strategy utilized Boolean operators to combine the selected terms. The operator AND was used to include intersecting elements, OR for the inclusion of any element in the sets, and NOT to exclude specific terms. The search equation implemented was as follows:

#1 prevalence [MeSH Terms]; #2 epidemiology [MeSH Terms]; #3 presence

#4 #1 OR #2 OR #3

#5 Trigger points [MeSH Terms]; #6 Myofascial pain syndrome [MeSH Terms]; #7 Myofascial trigger point; #8 Myofascial trigger points; #9 Trigger point

#10 #5 OR #6 OR #7 OR #8 OR #9

#11 Low back pain [MeSH Terms]; #12 Lumbar Vertebrae; #13 Lumbar; #14 Lumbar spine

#15 #11 OR #12 OR #13 OR #14

#16 #4 AND #10 AND #15

### 2.4. Eligibility Criteria

The inclusion criteria for this literature review were (1) descriptive, observational, or experimental studies published in English or Spanish that reported the prevalence of active or latent MTrPs in individuals with non-specific LBP; and (2) studies available in full text. Exclusion criteria included (1) review articles; (2) studies focusing on treatments without reporting data on MTrPs prevalence; (3) studies that did not clearly specify the procedures for identifying MTrPs; and (4) studies where the identification procedures for MTrPs did not adhere to the Delphi consensus guidelines [13,26]. No time filters were used and, therefore, all articles published at any time up to April 2025 were potentially eligible.

### 2.5. Methodological Quality Assessment

The methodological quality and risk of bias of the included studies were evaluated using a checklist derived from the Dutch Cochrane Centre, which was previously applied in a systematic review [21]. This tool analyzes seven criteria: (1) description of the patient group, (2) description of the control group, (3) selection bias, (4) exposure, (5) blinded measurement of exposure, (6) confounding factors, (7) outcomes. The quality assessment focused exclusively on study-level bias and did not evaluate individual results. Each criterion was rated as “+” if fulfilled, “−” if not fulfilled, or “0” if there was insufficient information to evaluate it. The final quality score was determined through a consensus meeting between the reviewers. Two of the authors conducted the risk-of-bias assessment for each included study. In cases of disagreement, a third researcher adjudicated to reach consensus.

### 2.6. Screening and Data Extraction

Articles retrieved from different databases were independently assessed by two authors with experience in review designs. First, duplicate articles were removed. Subsequently, titles and abstracts were reviewed to determine their eligibility. Finally, potentially eligible studies underwent full-text review. For an article to be included, both reviewers needed to reach a consensus. In cases of disagreement, a third experienced reviewer intervened to decide whether the study should be included or not.

Data extraction from each study was performed independently by two reviewers using a standardized form including details on participants, muscles evaluated, the number or percentage of active and/or latent MTrPs on each group, clinical characteristics of the participants, summary of results, and conclusions. Both reviewers were required to agree on each data point extracted. Any discrepancies were again resolved by a third reviewer who made the final decision.

## 3. Results

### 3.1. Study Selection

**Figure 1:**
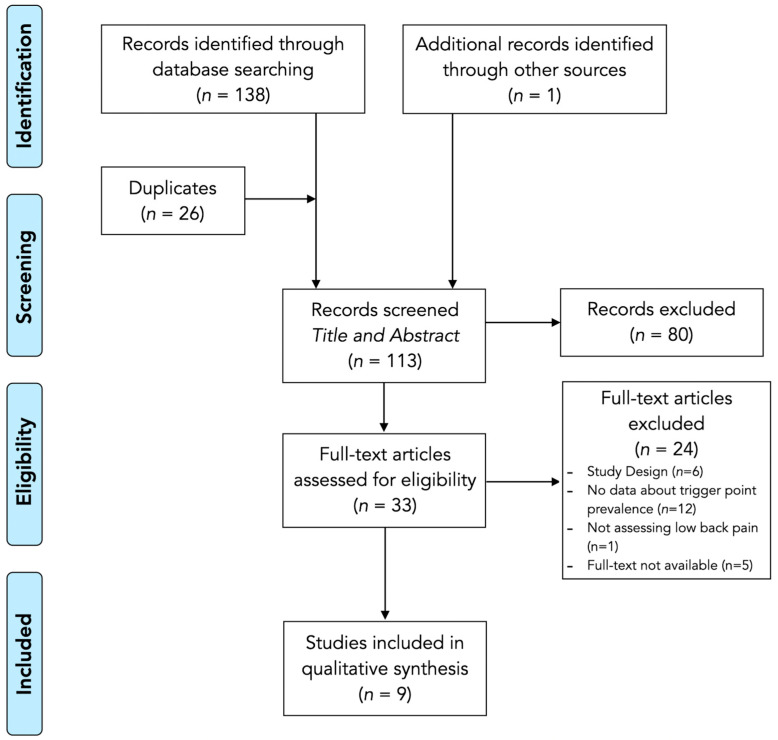
PRISMA flowchart diagram.

In an initial literature search, a total of 139 records were identified (*n* = 60 records were identified in PubMed, *n* = 32 in Cochrane, *n* = 46 in Web of Science, and *n* = 1 was manually identified). After an initial screening, duplicates (*n* = 26) were removed, resulting in a total of 113 non-duplicate records. Subsequently, after a first review of the title and abstract, studies that did not meet the described eligibility criteria (*n* = 80) were excluded due to the population assessed (individuals without a diagnosis of non-specific LBP). Among the remaining 33 studies analyzed in full text, 24 were excluded. Therefore, after the search, selection, identification, screening, eligibility assessment, and analysis process, nine articles were finally included in the review [16,17,27,28,29,30,31,32,33]. This procedure is detailed in the flowchart in Figure 1.

### 3.2. Study Characteristics

Of the nine studies finally selected, one was a clinical trial [29], six were observational studies [16,17,28,30,31,33], one was a quasi-experimental study [32], and one was a descriptive case series study [27]. These studies involved a total of 1187 participants (45.07% men). From this sample, *n* = 790 participants presented: acute LBP (lasting less than 2 months (*n* = 61) [28], chronic nonspecific LBP (*n* = 192) [17,27,29,30,31], and LBP associated with radiculopathy (*n* = 537) [16,30,32,33].

Table 1 details the characteristics of the included studies. Four studies differentiated between patients from asymptomatic control subjects [16,17,28,33], two studies assessed a single group of patients suffering from LBP without a comparator group [27,31], and the last study classified participants based on their LBP characteristics (radiating/non-radiating) [30].

In addition to the criteria for identification of MTrPs, four studies confirmed pain pressure sensitivity by using an algometer [16,27,29,33]. Regarding MTrP classification, three studies investigated both active and latent MTrPs [17,30,31], five analyzed only active MTrPs [16,27,28,32,33], and one focused exclusively on latent MTrPs [29].

The examined muscles included the quadratus lumborum [17,28,30], gluteus medius [16,17,27,28,29,30,31,33], gluteus minimus [16,17,30], piriformis [17,30], adductor longus [32], medial head of the gastrocnemius [32], tibialis anterior and posterior [32], short head of the biceps femoris [32], psoas [17], and lumbar iliocostalis [17].

### 3.3. Methodological Quality

The methodological quality assessment is detailed in Table 2. All studies scored from 4 [32] to 6 [29] points (mean score: 5.5; SD: 0.6 points).

All studies [16,17,27,28,29,30,31,32,33] distinguished between groups of patients with LBP (item 1), explicitly stated the eligibility criteria considering potential biases affecting the variability of MTrPs prevalence (item 3), provided detailed instructions for MTrP identification (item 4), and adequately reported the obtained results (item 7). On the other hand, only four studies include a control group (item 2) either by including pain-free healthy subjects or analyzing the contra-lateral symptomatic side in the group of LBP patients [17,28,30,33]. Additionally, only three studies implemented some level of blinding (item 5) [16,29,31], and four studies controlled for confounding factors (item 6) by measuring pressure pain thresholds at MTrP locations [16,27,29,33].

Overall, studies with higher methodological quality stood out for providing a comprehensive description of patient groups and a well-documented report of procedures, while aspects such as blinding and confounder control were less considered, limiting the methodological quality of several studies.

### 3.4. Results Synthesis: Prevalence of Active Trigger Points

Table 3 summarizes the prevalence of active MTrPs among individuals with radiating LBP and non-radiating LBP. Among patients with LBP, the prevalence of active MTrPs in the quadratus lumborum ranged from 30% to 55% [17,28,30]. Additionally, subjects with LBP showed a 45% prevalence of active MTrPs in the quadratus lumborum on the less painful side [17].

The gluteal region had a 76.4% prevalence of MTrPs in symptomatic patients, compared to 1.9% in the control group [33]. Another study analyzing different topographical regions demonstrated that the superolateral quadrant of the gluteus medius region had the highest prevalence of MTrPs (74.1%) [16].

In studies analyzing specific gluteal muscles, the gluteus medius showed MTrP prevalence ranging from 34% [28] to 45% [31] in patients with LBP. Other studies reported a prevalence of 35% on the painful side, with an even higher percentage (38%) on the contralateral side [17,30]. The gluteus minimus presented an MTrP prevalence of 5% on the less painful side and 12% on the more painful side [17], with another study reporting 42% prevalence [30]. The piriformis had a 42% prevalence of MTrPs in patients with LBP [30], with 35% on the painful side and 28% on the less painful side [17]. Finally, the psoas had a 5% MTrP prevalence on the less painful side and 10% on the more painful side [17].

The lumbar iliocostalis had an MTrP prevalence of 33% on the less painful side and 38% on the more painful side [17].

The only study that analyzed different MTrP prevalences within the same muscle in patients with low back pain was conducted by Salom-Moreno et al. [27]. This study focused on the gluteus medius, reporting an average of 5.6 MTrPs, with posterior and superior fibers being the most affected (93%), followed by anterosuperior and middle fibers (77%). The lowest prevalence was found in inferior fibers, with specific points showing prevalence rates of 62%, 54%, 46%, and 39% [27].

### 3.5. Results Synthesis: Prevalence of Latent Trigger Points

The prevalence of latent MTrP for each muscle is also summarized in Table 3. In one of the studies, the gluteus medius muscle showed a prevalence of 74% for latent MTrPs in patients with low back pain [33]. In another study, the prevalence was 12% on the painful side and 17% on the less painful side, while the control group presented a prevalence of 5% on both the dominant side and the non-dominant side [17].

The quadratus lumborum muscle showed a prevalence of 14% for MTrPs on the painful side and 17% on the less painful side in symptomatic patients, while in the control group, the prevalence was 0% on the dominant side and 10% on the non-dominant side [17]. The piriformis muscle had an MTrP prevalence of 22% on the PS and 19% on the less painful side in symptomatic patients, while in the control group, the prevalence was 0% on the dominant side and 7% on the non-dominant side. The psoas muscle in symptomatic patients showed an MTrP prevalence of 26% on the painful side and 36% on the less painful side, whereas in the control group, the prevalence was 19% on the dominant side and 26% on the non-dominant side [17]. Finally, the lumbar iliocostalis muscle had an MTrP prevalence of 19% on both the painful side and the less painful side in the patient group. In the control group, the prevalence was 0% on the painful side and 5% on the non-dominant side [17].

## 4. Discussion

This systematic review aimed to detail the prevalence of active and latent MTrPs focusing on patients with LBP. The gluteal complex and the quadratus lumborum emerged as the most frequently assessed regions. Overall, active MTrPs were more prevalent in the gluteal area of patients with radiating LBP, whereas those with non-radiating LBP exhibited higher rates of active MTrPs in the quadratus lumborum. However, these broad trends should be interpreted with caution, as prevalence figures can reverse when individual muscles or specific subdivisions within each region are examined in detail. In addition, although data is available for active MTrP located at the psoas, iliocostalis, and lower extremity muscles in patients with non-radiating LBP, no data is available for patients with radiating LBP. No data are available on latent MTrP prevalence in patients with radiating LBP. However, studies consistently report a higher burden of latent trigger points in non-radiating LBP compared with asymptomatic controls.

The literature underscores the clinical relevance of MTrPs and are consistent with the findings of this review, with studies reporting a prevalence of myofascial pain syndrome as high as 93% in patients attending pain clinics [34]. Given that previous systematic reviews and meta-analyses support interventions targeting MTrPs (such as dry needling, which has been demonstrated as an effective and safe intervention for reducing pain intensity and improving functional disability in this population [34,35,36,37] with effects comparable or superior to treatments such as acupuncture, laser therapy, or conventional physiotherapy), it is necessary to establish guidelines on which muscles exhibit the highest number of MTrPs.

The examination of MTrPs requires significant manual dexterity and knowledge of over 300 described pain maps [38,39]. Since this process is time-consuming and considering the low rate of success for the identification of certain MTrPs based on referred pain patterns [40], it is essential to define which MTrPs are most prevalent to guide clinicians toward the locations that most frequently reproduce the patient’s symptoms, thereby reducing the time required for diagnosis.

Following a comprehensive literature review, nine studies met the eligibility criteria with an average methodological quality score of 5.0 out of 7 (indicating a moderate to high level of rigor). Based on these reports, the most relevant findings were the considerable variability in the prevalence of active and latent MTrPs for this population. The analysis of MTrP prevalence in relation to low back pain etiology revealed significant differences between patients with nonspecific and radicular pain, as well as variations in the most affected muscles according to symptom chronicity. In patients with nonspecific low back pain, MTrPs are highly prevalent [41], with studies indicating their presence in more than 80% of cases [16,17,27,28,29,30,31,32,33]. Within this group, the most commonly affected muscles include the quadratus lumborum and gluteus medius, likely due to their stabilizing role in the lumbar region and their susceptibility to mechanical overload from postural alterations and dysfunctional movement patterns [42,43,44].

Conversely, in patients with lumbosacral radiculopathy, the presence of MTrPs in the gluteal musculature reached up to 76.4% in the gluteus medius [33]. This finding suggests that irritation of lumbar nerve roots may contribute to the development of MTrPs in muscles innervated by these structures, either through a reflex mechanism or biomechanical compensations secondary to neuromuscular dysfunction [45].

The chronicity of LBP also appears to influence the distribution and activation of MTrPs. In patients with chronic nonspecific pain, the prevalence of active MTrPs is higher than that of latent MTrPs, particularly in the quadratus lumborum, lumbar iliocostalis, and gluteus medius muscles [17]. These muscles not only exhibit the highest number of active MTrPs, but the quantity of active MTrPs within them correlates directly with pain intensity and patient disability. This relationship may be mediated by central sensitization and the perpetuation of dysfunctional motor patterns, leading to continuous activation of MTrPs [46].

Regarding specific localization within a muscle, MTrPs in the gluteus medius are most frequently found in the posterior and superior fibers, followed by the anterosuperior and middle fibers [27]. This distribution may be related to the greater biomechanical load borne by these regions during gait and pelvic stabilization, predisposing them to myofascial dysfunction [47,48]. Finally, in the context of low back pain with radiation, the presence of MTrPs in the gluteal region may serve as a useful clinical marker for differentiating between radicular and non-radicular pain. One study found that MTrPs in the superolateral quadrant of the gluteus exhibit 91.4% specificity in predicting radicular involvement, suggesting that their assessment could reduce the need for more invasive diagnostic procedures [16].

However, these findings should be interpreted with caution, as despite following widely accepted recommendations for MTrP identification, these guidelines rely on manual palpation, a procedure inherently subjective and with controversial inter-examiner reliability [49]. One study included in the review reported an overall inter-examiner agreement of 84% in identifying MTrPs in patients with radicular low back pain, with a mean kappa coefficient of 0.66, indicating substantial agreement in detecting these points. However, reproducibility varied by muscle, with kappa values ranging from 0.42 for the quadratus lumborum to 0.83 for the gluteus medius [30]. These results suggest that muscles that are more accessible to palpation demonstrate higher reliability indices than those with indirect or less accessible anatomical landmarks, and this might be the reason explaining the low MTrP prevalence in deep muscles such as the psoas or gluteus minimus.

Another relevant aspect is the difficulty in determining the precise localization of MTrPs. It was found that 57% of MTrPs identified by one examiner were not corroborated by another within a 50 mm margin, indicating considerable variability in their precise location [30]. This finding underscores the need for complementary, more objective methods, such as ultrasound elastography or electromyography, to enhance reliability in MTrP detection and mitigate the subjective bias associated with manual evaluation [42].

Moreover, discrepancies were also noted in the identification of the number of MTrPs per patient, with differences ranging from 0.8 to 6.4 points between examiners [30]. This implies that, in clinical practice, two professionals might reach different conclusions regarding the quantity and localization of MTrPs, potentially influencing therapeutic decisions and treatment planning.

### 4.1. Future Resarch

While pain duration and intensity are likely to modulate both the prevalence and anatomical distribution of MTrPs [17], our ability to explore these relationships quantitatively is limited by inconsistent reporting. Future prevalence studies should systematically record and stratify participants by standardized pain-duration categories (e.g., acute ≤6 weeks, subacute 6–12 weeks, chronic >12 weeks) and validated pain-intensity scales. Such uniform reporting would enable subgroup analyses or meta-regressions to determine whether longer-standing or more severe pain is associated with higher MTrP prevalence or specific muscular patterns.

### 4.2. Limitations

This systematic review has some limitations. First, only studies published in English or Spanish were considered, leading to the exclusion of relevant research in other languages. Additionally, the scarcity of studies investigating the presence of MTrPs in individuals with low back pain, along with the lack of a comparison group in the different studies, means that the results of this review should be interpreted with caution. To more robustly support the presence of MTrPs in low back pain, further studies with appropriate comparisons are needed.

## 5. Conclusions

This systematic review highlights the significant prevalence of MTrPs in patients with low back pain, emphasizing their potential role in symptom generation and clinical management. The findings suggest that active and latent MTrPs are frequently present in muscles such as the quadratus lumborum, gluteus medius, and iliocostalis, with variations in prevalence depending on pain chronicity and etiology. Patients with lumbosacral radiculopathy tend to exhibit a higher prevalence of MTrPs in the gluteal region, potentially linking MTrP development to neurogenic mechanisms. However, despite the widespread use of manual palpation for MTrP identification, its subjectivity and limited inter-examiner reliability remain important considerations, underscoring the need for more objective diagnostic tools. Furthermore, the heterogeneity of study designs, variability in diagnostic criteria, and lack of standardization in prevalence reporting limit the generalizability of these findings. Future research should aim to refine MTrP diagnostic protocols and explore their pathophysiological significance to enhance therapeutic interventions for LBP.

## Figures and Tables

**Table 1:** Characteristics of the studies included.

Reference	Study Design	Demographic Characteristics	Clinical Characteristics	Complementary MTrP Identification Procedures	Muscles Assessed
Adelmanesh et al., 2015 [33]	Case–Control	*n* = 271 Males 43.2% Females 56.8%	Radiating Low Back Pain	Pain Pressure Thresholds	Gluteus region (no muscles specified)
Adelmanesh et al., 2016 [16]	Diagnostic accuracy	*n* = 185 Males 47% Females 53%	Radiating Low Back Pain and Non-Radiating Low Back Pain	Pain Pressure Thresholds	Gluteus region (no muscles specified)
Álvarez-Delgado et al., 2022 [29]	Randomized Clinical Trial	*n* = 87 *n* = 80 with MTrPs *n* = 7 without MTrPs	Non-Radiating Low Back Pain	Pain Pressure Thresholds	Gluteus medius
Carroll et al., 2022 [31]	Observational	*n* = 42 Males 17% Females 83%	Non-Radiating Low Back Pain	No	Quadratus lumborum
Holm-Jensen et al., 2020 [30]	Inter-examiner reliability	*n* = 32 Males 53% Females 47%	Radiating Low Back Pain and Non-Radiating Low Back Pain	No	Quadratus lumborum Gluteus medius Gluteus minimus Piriformis
Iglesias-González et al., 2013 [17]	Observational	*n* = 42 Males 50% Females 50%	Non-Radiating Low Back Pain	No	Quadratus lumborum Lumbar iliocostalis Psoas Piriformis Gluteus medius Gluteus minimus
Hua et al., 1994 [28]	Case–Control	*n* = 124 Males 52.4% Females 47.6%	Non-Radiating Low Back Pain	No	Quadratus lumborum Gluteus medius
Saeidian et al., 2014 [32]	Quasi-experimental	*n* = 98 *n* = 64 with MTrPs *n* = 34 without MTrPs	Radiating Low Back Pain	No	Adductor longus Medial head of the gastrocnemius Tibialis anterior and posterior Short head of the biceps femoris Lumbar paraspinal muscles
Salom-Moreno et al., 2015 [27]	Case series	*n* = 13 Males 23.1% Females 76.9%	Non-Radiating Low Back Pain	Pain Pressure Thresholds	Gluteus medius

**Table 2:** Methodological quality results (Dutch Cochrane Centre tool).

Reference	Patient Group Description	Control Group Description	Selection Bias	Exposure	Blinded Measurement of Exposure	Confounders	Results	Score
Adelmanesh et al., 2015 [33]	1	1	1	1	0	1	1	6/7
Adelmanesh et al., 2016 [16]	1	0	1	1	1	1	1	6/7
Álvarez-Delgado et al., 2022 [29]	1	0	1	1	1	1	1	6/7
Carroll et al., 2022 [31]	1	0	1	1	1	0	1	5/7
Holm-Jensen et al., 2020 [30]	1	1	1	1	0	0	1	5/7
Iglesias-González et al., 2013 [17]	1	1	1	1	0	0	1	5/7
Hua et al., 1994 [28]	1	1	1	1	0	0	1	5/7
Saeidian et al., 2014 [32]	1	0	1	1	0	0	1	4/7
Salom- Moreno et al., 2015 [27]	1	0	1	1	0	1	1	5/7

**Table 3:** Qualitative synthesis of the results categorized by muscle and trigger point classification.

Active Myofascial Trigger Points		Latent Myofascial Trigger Points			References
Radiating Low Back Pain	Non-Radiating Low Back Pain	Radiating Low Back Pain	Non-Radiating Low Back Pain	Asymptomatic Subjects	
Quadratus lumborum					
-	36%	-	-	-	Hua et al., 1994 [28]
30%	-	-	-	-	Holm-Jensen et al., 2020 [30]
-	55% ^a^–45% ^b^	-	14% ^a^–17% ^b^	0% ^c^–10% ^d^	Iglesias-González et al., 2013 [17]
Gluteal region (no specific muscles reported)					
76.4%	-	-	-	-	Adelmanesh et al., 2015 [33]
74.1%	8.5%	-	-	-	Adelmanesh et al., 2016 [16]
Gluteus medius					
-	-	-	91%	-	Álvarez-Delgado et al., 2022 [29]
-	39–93%	-	-	-	Salom-Moreno et al., 2015 [27]
-	43–45%	-	74%	-	Carroll et al., 2022 [31]
-	34%	-	-	-	Hua et al., 1994 [28]
35%	-	-	-	-	Holm-Jensen et al., 2020 [30]
-	35% ^a^–38% ^b^	-	12% ^a^–17% ^b^	5%	Iglesias-González et al., 2013 [17]
Gluteus minimus					
-	12% ^a^–5% ^b^	-	7% ^a^–12% ^b^	12 ^c^–10% ^d^	Iglesias-González et al., 2013 [17]
42%	-	-	-	-	Holm-Jensen et al., 2020 [28]
Piriformis					
42%	-	-	-	-	Holm-Jensen et al., 2020 [30]
-	35% ^a^–28% ^b^	-	22% ^a^–19% ^b^	0% ^c^–7% ^d^	Iglesias-González et al., 2013 [17]
Psoas					
-	10% ^a^–5% ^b^	-	26% ^a^–36% ^b^	19% ^c^–26% ^d^	Iglesias-González et al., 2013 [17]
Iliocostalis					
-	38% ^a^–33% ^b^	-	19%	0% ^c^–5% ^d^	Iglesias-González et al., 2013 [17]
Lower Extremity (no specific muscles reported)					
-	65% ^a^	-	-	-	Saeidian et al., 2014 [32]

^a^: Painful side; ^b^: Non-painful side; ^c^: Dominant side; ^d^: Non-dominant side.

## Data Availability

The original contributions presented in this study are included in the article. Further inquiries can be directed to the corresponding author(s).

## Abbreviations

The following abbreviations are used in this manuscript:

LBP	Low back pain
MeSH	Medical Subject Headings
MTrP	Myofascial trigger point
PICO	Population, Intervention, Comparison, Outcome
PRISMA	Preferred Reporting Items for Systematic Reviews and Meta-Analyses
